# How to survive pig farming: Mechanism of SCC*mec* element deletion and metabolic stress adaptation in livestock-associated MRSA

**DOI:** 10.3389/fmicb.2022.969961

**Published:** 2022-11-23

**Authors:** Charlotte Huber, Silver A. Wolf, Wilma Ziebuhr, Mark A. Holmes, Julia Assmann, Antina Lübke-Becker, Andrea Thürmer, Torsten Semmler, Julian Brombach, Astrid Bethe, Markus Bischoff, Lothar H. Wieler, Lennard Epping, Birgit Walther

**Affiliations:** ^1^Advanced Light and Electron Microscopy (ZBS4), Robert Koch Institute, Berlin, Germany; ^2^Genome Sequencing and Genomic Epidemiology (MF2), Robert Koch Institute, Berlin, Germany; ^3^Institute for Molecular Infection Biology, University of Würzburg, Würzburg, Germany; ^4^Department of Veterinary Medicine, University of Cambridge, Cambridge, United Kingdom; ^5^Institute of Microbiology and Epizootics, Freie Universität Berlin, Berlin, Germany; ^6^Institute of Medical Microbiology and Hygiene, Saarland University, Homburg, Germany; ^7^Methodology and Research Infrastructure, Robert Koch Institute, Berlin, Germany

**Keywords:** methicillin resistant *Staphylococcus aureus*, livestock associated, SCC*mec*, transcriptome analysis, recombination, deletion, ammonium, manure (litter)

## Abstract

Previous research on methicillin susceptible *Staphylococcus aureus* (MSSA) belonging to livestock-associated (LA-) sequence type (ST) 398, isolated from pigs and their local surroundings, indicated that differences between these MSSA and their methicillin resistant predecessors (MRSA) are often limited to the absence of the staphylococcal cassette chromosome *mec* (SCC*mec*) and few single nucleotide polymorphisms. So far, our understanding on how LA-MRSA endure the environmental conditions associated with pig-farming as well as the putative impact of this particular environment on the mobilisation of SCC*mec* elements is limited. Thus, we performed in-depth genomic and transcriptomic analyses using the LA-MRSA ST398 strain IMT38951 and its methicillin susceptible descendant. We identified a mosaic-structured SCC*mec* region including a putative replicative SCC*mec*Vc which is absent from the MSSA chromosome through homologous recombination. Based on our data, such events occur between short repetitive sequences identified within and adjacent to two distinct alleles of the large cassette recombinase genes C (*ccr*C). We further evaluated the global transcriptomic response of MRSA ST398 to particular pig-farm associated conditions, i.e., contact with host proteins (porcine serum) and a high ammonia concentration. Differential expression of global regulators involved in stress response control were identified, i.e., ammonia-induced alternative sigma factor B-depending activation of genes for the alkaline shock protein 23, the heat shock response and the accessory gene regulator (*agr*)-controlled transcription of virulence factors. Exposure to serum transiently induced the transcription of distinct virulence factor encoding genes. Transcription of genes reported for mediating the loss of methicillin resistance, especially *ccr*C, was not significantly different compared to the unchallenged controls. We concluded that, from an evolutionary perspective, bacteria may save energy by incidentally dismissing a fully replicative SCC*mec* element in contrast to the induction of *ccr* genes on a population scale. Since the genomic SCC*mec* integration site is a hot-spot of recombination, occasional losses of elements of 16 kb size may restore capacities for the uptake of foreign genetic material. Subsequent spread of resistance, on the other hand, might depend on the autonomous replication machinery of the deleted SCC*mec* elements that probably enhance chances for reintegration of SCC*mec* into susceptible genomes by mere multiplication.

## Introduction

Since the mid-2000s, sequence type (ST)398 and its closely related descendants represent the predominant clonal complex (CC) of livestock-associated methicillin resistant *Staphylococcus aureus* (LA-MRSA) throughout Europe (Butaye et al., 2016). ST398 LA-MRSA most likely originate from a lineage of human methicillin susceptible *S. aureus* (MSSA) and have since adapted to livestock (particularly to pig farming) *via* acquisition of resistance genes against tetracycline and beta-lactam antibiotics (Price et al., 2012). ST398 LA-MRSA are also capable of causing infections in humans (Williamson et al., 2014; Sieber et al., 2019; Lu et al., 2021), a problem that is of particular concern across regions with high pig farming density (Köck et al., 2009; Vanderhaeghen et al., 2010; Gómez et al., 2020). Indeed, some MRSA are prone to cross species barriers (Sheppard et al., 2018) and are capable of rapid adaptation to different habitats and environmental conditions (Larsen et al., 2022). Adaptation processes were mainly studied on the genomic level and include either changes of the core genome (Huber et al., 2020) or an uptake of mobile genetic elements (MGEs; Mccarthy and Lindsay, 2013; Walther et al., 2018; Sieber et al., 2019; Huber et al., 2020; Leinweber et al., 2021).

Genome alterations also affect the staphylococcal cassette chromosome *mec* (SCC*mec*) including its *mec* complex which confers broad-spectrum β-lactam resistance in *S. aureus* and whose spontaneous loss was reported for ST398 isolates (Chlebowicz et al., 2010; Gómez et al., 2020). SCC*mec* elements represent large genomic islands of various sizes and compositions, which are inserted into the highly conserved region between the *rlm*H (previously *orfX*) and *dus* (previously *orf*Y) genes of staphylococcal chromosomes (Semmler et al., 2016). Loss of SCC*mec* elements can either result from homologous recombination (HR) events (involving the recombination apparatus of the cell) or through precise excision of the element from its integration site. The latter process is driven by the SCC*mec*-encoded site-specific serine recombinases (i.e., *ccr*A/B or *ccr*C) which also mediate integration of the element into the *rlm*H gene (Hanssen and Ericson Sollid, 2006; Chlebowicz et al., 2010; Wang and Archer, 2010; Liu et al., 2017; Gómez et al., 2020).

Interestingly, loss of SCC*mec* is thought to provide an advantage for *S. aureus* in challenging environments, for example when exposed to antibiotics (Noto et al., 2008) and/or the host immune system (Read et al., 2018; Tickler et al., 2020); and in LA-MRSA, the specific environmental conditions surrounding pig farming were proposed as factors that might facilitate deletion of the element (Nübel et al., 2010; Vandendriessche et al., 2014).

In industrial pig farming, the toxic gas ammonium hydroxide (NH_3_) is released from manure during (bacterial) deamination of proteins fed to the animals. The gas subsequently accumulates in the air of the barn and, due to its high solubility in fluids, it is harmful to airway mucosal surfaces of pig farm workers and exposed pigs (Urbain et al., 1996; Wang et al., 2020) Thus, it is unavoidable that MRSA colonizing moist mucosal surfaces of the anterior nostrils of pigs are exposed to increased concentrations of ammonia. Therefore, exposure to high concentrations of ammonia is a condition commonly encountered by LA-MRSA ST398, and a recent study demonstrated that these bacteria indeed survive under such conditions (Astrup et al., 2021).

Regarding cytosolic pH, ammonia is the preferred nitrogen source of most microorganisms, and once in the cell, is directed to glutamine synthesis (Gruswitz et al., 2007). However, information on ammonia-induced (metabolic) gene expression patterns in LA-MRSA are currently scarce. Aside from exposure to the harsh barn environment, LA-MRSA remain associated with pigs as their primary host, which illustrates another condition the bacteria are required to adapt to.

Taking these different environmental conditions into account, the aims of this study were: (i) to reconstruct the region downstream of *rlm*H in an ST398 MRSA and its methicillin susceptible descendant (MSSA) in order to reveal the mechanisms involved in the loss of SCC*mec*; (ii) to evaluate the global transcriptomic response of MRSA ST398 exposed to particular LA environmental conditions, i.e., contact with host proteins (porcine serum) and enhanced ammonia concentrations, with a special focus on (iii) transcription levels of genes previously reported as being associated with the acquisition or loss of SCC*mec* elements.

## Materials and methods

### Bacterial strains

Strain MRSA IMT38951, isolated from a nose swab of a pig in 2016, and its isogenic MSSA descendant IMT38951_42 (Busche et al., 2018) were used throughout this study. The MSSA isolate was discovered during serial-dilutions of overnight grown broth cultures [Mueller-Hinton (MH) bouillon (Becton Dickinson, Heidelberg, Germany)] of IMT38951. Presence and absence of methicillin resistance was determined according to CLSI standards (Clinical and Laboratory Standards Institute, 2013, 2014) and verified by PCR (Merlino et al., 2002). MSSA IMT38951_42 was enclosed to comparatively analyze the chromosomal integration site downstream of *rlm*H (Boundy et al., 2013), as described previously (Chlebowicz et al., 2010; Wang et al., 2017; Wu et al., 2018; Gómez et al., 2020).

### DNA isolation, whole-genome sequencing and assembly

Genomic bacterial DNA was extracted using the QIAamp DNA Mini Kit (QIAGEN, Venlo, Netherlands) according to the manufacturer’s instructions. Isolates were pre-incubated with 0.1 mg/ml Lysostaphin and 0.1 mg/ml Proteinase K (both from Sigma-Aldrich, St. Louis, Missouri, United States). NanoDrop Spectrophotometer (Thermo Fisher Scientific, United States) as well as the Qubit 2.0 Fluorometer (Invitrogen, United States) were used to evaluate the DNA quality and quantity, respectively.

Whole-genome sequencing (WGS) was performed using an Illumina MiSeq which resulted in 300 bp paired-end reads with an obtained coverage >90X. Furthermore, size selection was conducted on the DNA using a 10 K Blue Pippin kit for additional sequencing with the Single Molecule Real-Time (SMRT) Sequencing Technology on a PacBio RS II by GATC Biotech AG (Konstanz, Germany). PacBio raw data was assembled by Flye v2.8.2-b1689 (Kolmogorov et al., 2019). Adapter-trimmed Illumina short reads were used for assembly correction utilizing the polishing procedure of unicycler v0.4.8 (Wick et al., 2017). *De novo* assembled genomes were annotated using Prokka v1.14.6 (Seemann, 2014). Both genomes were comparatively investigated with respect to single nucleotide polymorphisms (SNPs), insertions and deletions by read mapping utilizing botwie2 v2.3.0 (Langmead and Salzberg, 2012). Putative sequence variants were defined as divergent nucleotides with a minimum coverage of 10X and an allele frequency of >80%. ResFinder-4.1 (Zankari et al., 2012; Bortolaia et al., 2020) was used to identify resistance genes, and further genomic sites of interest were investigated using Geneious 11.1.5 (Biomatters Ltd., Australia; Kearse et al., 2012). PHASTER (Arndt et al., 2016) was utilized to identify putative (pro-) phages across both genomes.

### Selection of RNA Seq samples

Cells of IMT38951 were grown in 60 ml MH II and incubated overnight at 37°C and 250 rpm and, on the following day, diluted to an OD_600_ of 0.1 in 100 ml of fresh MH II and further incubated at 37°C and 250 rpm.

Based on previous research (Anderson et al., 2010; Malachowa et al., 2011; Cardile et al., 2014; Pinon et al., 2015) we used an ammonia concentration of 0.3% (Th. Geyer, Renningen, Germany), 10% porcine serum (Innovative Research Inc., Peary Court Novi, Michigan, United States) as well as a combination of both in order to mimic environmental conditions associated with pig-farming. Sampling time points were selected based on results of Stojanov et al. (2015) on SCC*mec* excision and the major regulative activity of the accessory gene regulator (*agr*)-system (Wang and Muir, 2016; Huber et al., 2020). At an OD_600_ of 0.4, the broths were supplemented with either porcine serum (10%), ammonia (0.3%), or both. To ensure the viability of the bacteria in the supplemented media, the viable plate count was determined every 15 min (0–90 min after media supplementation). Media from controls were not supplemented.

A previously published protocol (Lavoie and Summers, 2018) was used to select the samples for RNA sequencing to guarantee overall comparability. Briefly, for each of the experiments, seven biological replicates were initially grown alongside seven corresponding controls. The OD_600_ was determined for each set of samples and controls (Supplementary Table 1). Then, three out of the seven samples that exhibited the most similar OD_600_ values per growth condition and time point (10 and 60 min after media supplementation) were selected for subsequent RNA isolation (*n* = 18 samples). In addition, the three most similar control samples per growth condition and time point were selected as well (*n* = 18; written in bold in Supplementary Table 1). Each aliquot was centrifuged at 20,000 g and 4°C for 3 min. The supernatant was aspirated, and the pellet was immediately frozen at −80°C following previously published protocols (Mäder et al., 2016; Lavoie and Summers, 2018).

### RNA sequencing and differential transcriptomic analysis

In total, 36 cell pellets (18 samples and 18 associated controls) were shipped to LGC Genomics GmbH (Berlin, Germany), where the RNA was isolated using the RNASnap method (Stead et al., 2012). RNA sequencing was performed on an Illumina NextSeq 500/550 V2, resulting in one channel paired-end reads. Details of the company’s standard protocols for quality control, RNA extraction and rRNA depletion using Ribo-Zero (Epicentre Biozym, Hessisch Oldendorf, Germany) are available online.^1^ cDNA synthesis, library generation, indexing and cluster generation were performed using Illumina technology (TruSeq RNA Sample Preparation Kit v2).

SCORE v1.0.2 (Wolf et al., 2020) was used to perform differential RNA sequencing analysis by comparing the transcriptomic profiles of selected challenges (ammonia, porcine serum or the combination of them) with control samples, resulting in the identification of differentially expressed genes (DEGs) across the sample set. Initial analysis steps including read preprocessing (trimming of low-quality ends with Phred score < 20), mapping to the genomic reference, transcript quantification (genes below 10 counts were discarded), statistical identification of differentially expressed genes between sample groups (corrected *p* ≤ 0.05), overrepresentation analysis of associated Gene Ontologies (GO) and visualization of affected Kyoto Encyclopedia of Genes and Genomes (KEGG) pathways were performed within SCORE. An additional fold change filter (log2FC > 1) was applied to ensure biological relevancy of the DEGs (Slany et al., 2017). Genes not fulfilling these criteria were counted as not being differentially expressed between conditions. The resulting expression tables were subsequently merged and additionally characterized using CD-Search v3.18 (Marchler-Bauer and Bryant, 2004) and eggNOG-mapper v2.0.5 (Huerta-Cepas et al., 2019). Principal component analysis (PCA) was performed based on normalized transcripts per million (TPM) expression values to assess inter-sample distances (Scholes and Lewis, 2020).

## Results

### Reconstruction of the SCC*mec* integration site downstream of *rlm*H revealed the loss of a complete SCC*mec*Vc element harbouring a replication machinery

**Figure 1:**
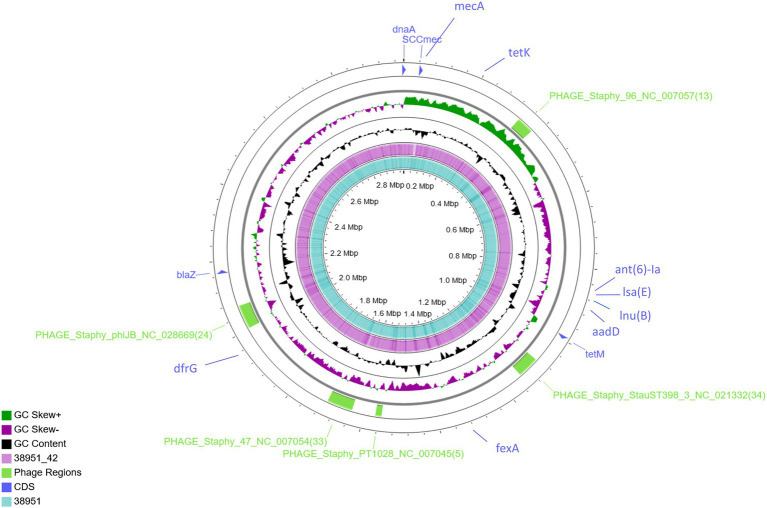
Genome-wide comparison of MRSA ST398 (IMT38951) and its descendant MSSA (IMT38951_42). A combinatorial approach including long reads (PacBio) and short reads (Illumina sequencing) allowed precise reconstruction of both genomes. Five regions harbouring phages or phage-associated genes identified using PHASTER are indicated in green. Location of resistance genes identified using ResFinder 2.1 and the SCC*mec* intergration site are indicated in blue. Comparative genomics determined a difference of 10 single nucleotide polymorphisms in total (excluding phage regions).

To allow determination of the precise genomic sites flanking the loss of a *mec*A-containing sequence in MSSA IMT38951_42, a finished whole-genome reference sequence was required. PacBio long reads were utilized to assemble the draft genomes using Flye, resulting in a coverage of >335-times and > 331-times. Short-read polishing of the genomes was performed, providing an additional 161X and 187X sequence coverage to the reconstructed genomes of IMT38951 (MRSA) and IMT38951_42, respectively (Figure 1).

Further comparative WGS analysis revealed an overall genome size of 3,022,864 bases for IMT38951 and 3,011,968 bases for IMT38951_42. The former contains 2,896 coding sequences (CDS), 19 rRNA and 61 tRNAs, while the latter harbors 30 CDS less according to the annotation. Initial strain typing of IMT38951 and IMT38951_42 assigned both isolates to LA-ST398 and *spa* type t12359. In addition to the *mec*A gene present in IMT38951, both genomes harbour a similar set of further resistance genes [*aad*D, *fex*A, *bla*Z, *dfr*G, *tet*(K), *tet*(M), *lnu*(B) and *lsa*(E); Figure 1]. Prediction of prophages by PHASTER based on sequence similarity-based searches revealed three hits for complete phages, PHAGE_Staphy_47_NC_007054 (72.2 kb in size), PHAGE_Staphy_phiJB_NC_028669 (60 kb) and PHAGE_Staphy_StauST398_3_NC_021332 (66.9 kb) and two further incomplete phages of the lengths 16.6 kb (PHAGE_Staphy_PT1028_NC_007045) and 41.2 kb (PHAGE_Staphy_47_NC_007054), respectively (Figure 1). Both strains harbour a small plasmid of 5,359 bp size that resembles the *Staphylococcus hyicus* plasmid pKKS966 (99.82% identity, 73% coverage GenBank: FN677368.1) carrying the trimethoprim resistance gene *dfr*K (Kadlec et al., 2012).

**Figure 2:**
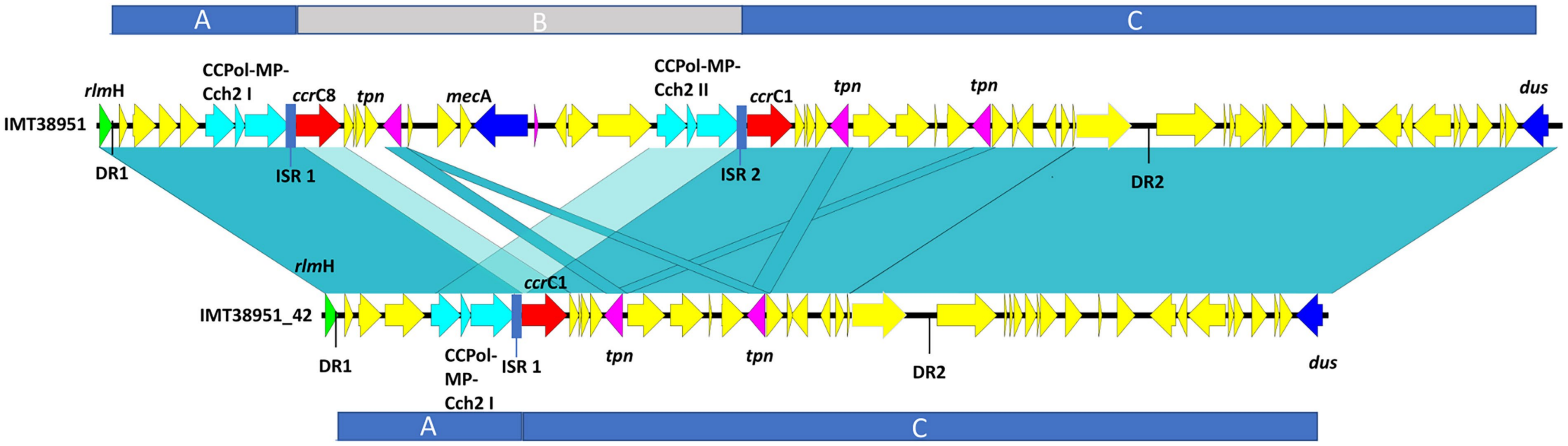
Sequence comparison of IMT38951 and IMT38951_42 from *rlm*H (previously: *orf*X) to *dus* (previously *orf*Y). Repetitive elements and homologues regions challenged the precise reconstruction of MRSA IMT38951 and its isogenic descendant lacking SCC*mec*Vc (region B). Since sequence identities of genes and intergenic spacer regions present in this region range from 98/99% (light blue-green) to 100% (blue-green), including sequences and genes that flank (regions A and C) the missing SCC*mec* element in IMT38951_42. DR, direct repeat of 15 bp length; CCPol-MP-Cch2, operon containing an A-family DNA polymerase (CCPol) together with a small protein lacking conserved domains (“middle protein,” MP) as well as a putative helicase (Cch2) (Bebel et al., 2020); *ccr*C, chromosomal cassette recombinase C; *tpn*, transposase associated with insertion element (IS) 431; ISR, intergenic spacer region of 224 bp length.

**Figure 3:**
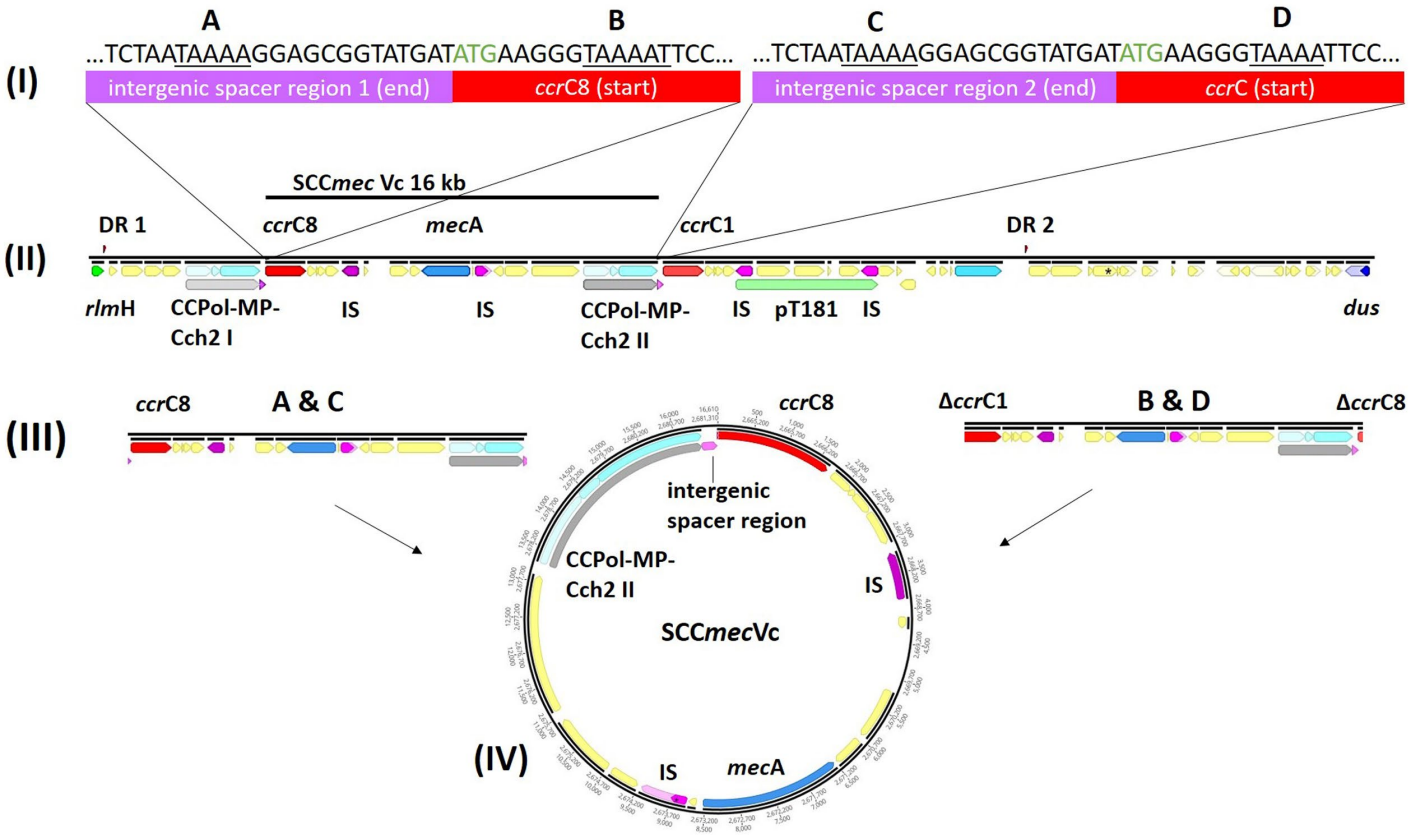
Tracking the loss of SCC*mec*Vc. MRSA ST398 IMT38951: Illustration of the SCC*mec* integration site downstream of *rlm*H. **(A)** Comparison of the terminal sequences of the intergenic sequences spacer region 1 (I) and 2 (III) and the initial basepairs of *ccr*C8 (II) and *ccr*C1 (IV) (start codon: green) revealed two possibilities of homologues recombination after deletion of SCC*mec:* A & C, intergenic regions 1 and 2; and B & D, *ccr*C8 and *ccr*C1. **(B)** The region between *rlm*H (previously “*orf*X“) and *dus* (previously *orf*Y) carries various repetitive DNA sequences. **(C)** Both recombination events would allow deletion of an identical and complete SCC*mec*Vc element. **(D)** Circular SCC*mec* element with putative functional recombinase *ccr*C8 and a CCPol-MP-Cch2 II complex (Bebel et al., 2020) conferring its autonomous replication capabilities. *ccr*, chromosomal cassette recombinase; IS, insertion sequence; DR, directs repeats associated with *ccr*-mediated integration and excision of SCC*mec* elements (Semmler et al., 2016).

Pairwise distance and relatedness (Figure 1) was calculated, revealing a total difference of 10 SNPs (excluding phage regions) and the lack of a 16 kb sequence downstream of the SCC*mec* integration site (*rlm*H) in IMT38951_42 (Figure 2). A closer inspection of the region revealed the loss of a complete SCC*mec*Vc element (Figure 2) comprising the *ccr*C8 type recombinase, the C2 *mec* complex and a recently described putative operon containing an A-family DNA polymerase (CCPol) together with a small protein lacking conserved domains (“middle protein,” MP) as well as a putative helicase (Cch2; referred to as “CCPol-MP-Cch2” I & II in Figures 2, 3), as recently described by Bebel et al. (2020). The region downstream of *rlm*H in IMT38951 includes a notable repertoire of homologues sequences, i.e., two distinct but closely related *ccr*C variants and two nearly homologues intergenic spacer regions of 224 bp length, four transposases (*tnp*) of insertion sequence (IS) element 431 (IS431) and two variants of the replication machinery CCPol-MP-Cch2 (Figure 2; Supplementary Table 2) (region *rlm*H – *dus*, highlighted in green). The data suggest that the deletion of SCC*mec*Vc occurred during HR between either *ccr*C8 and *ccr*C1 (first 48 bp are 100% identical) or the terminal 38 bp (identity: 100%) of the two 224 bp-comprising intergenic spacer regions upstream of the *ccr*C variants, respectively (Figure 3; Supplementary Figure 1). Both loci harbour short repetitive sequences (“TAAAA,” A & B and C & D in Figure 3) that provide at least two distinct possibilities for HR without any further sequence alterations apart from the loss of the SCC*mec* element within this region. Both intergenic spacer sequences present in the MRSA variant harbor the original (identical) promoter region of *ccr*C variants, including the 19 bp SOS box described by Liu et al. (Liu et al., 2017; Supplementary Figure 1). Interestingly, the deleted SCC*mec*Vc element still harbours its putative autonomous replication apparatus, since all loci involved (direct repeats, inverted repeats, origin of replication, CCPol-MP-Cch2 II) remain completely unaffected by the proposed recombination event (Supplementary Figure 1).

A *ccr*-mediated integration and excision, on the other hand, is known to change the number of 15 bp DR in the region downstream of *rlm*H (Semmler et al., 2016). Here, the total number and location of the two distinct 15 bp repeats present in the MSSA descendant is unchanged compared to its MRSA predecessor (Figure 2), arguing against recombinase driven loss of SCC*mec*Vc.

Further sequence comparison with the type V SCC*mec* (GenBank: AB512767) genes and intergenic regions of a strain (TSGH17) belonging to another livestock-associated genetic background (ST59), used by Bebel et al. (2020) to describe the CCPol-MP complex and their Cch2 helicases, revealed a sequence identity of 94.9% and 80 SNPs, two larger insertions/deletions of 40 bp and 1,275 bases with 4 single insertions/deletions proving continuous evolvement of the region downstream of *rlm*H.

### Exposure of ST398 to porcine serum and ammonia alters activity of regulatory pathways and genes involved in environmental adaptation

**Figure 4:**
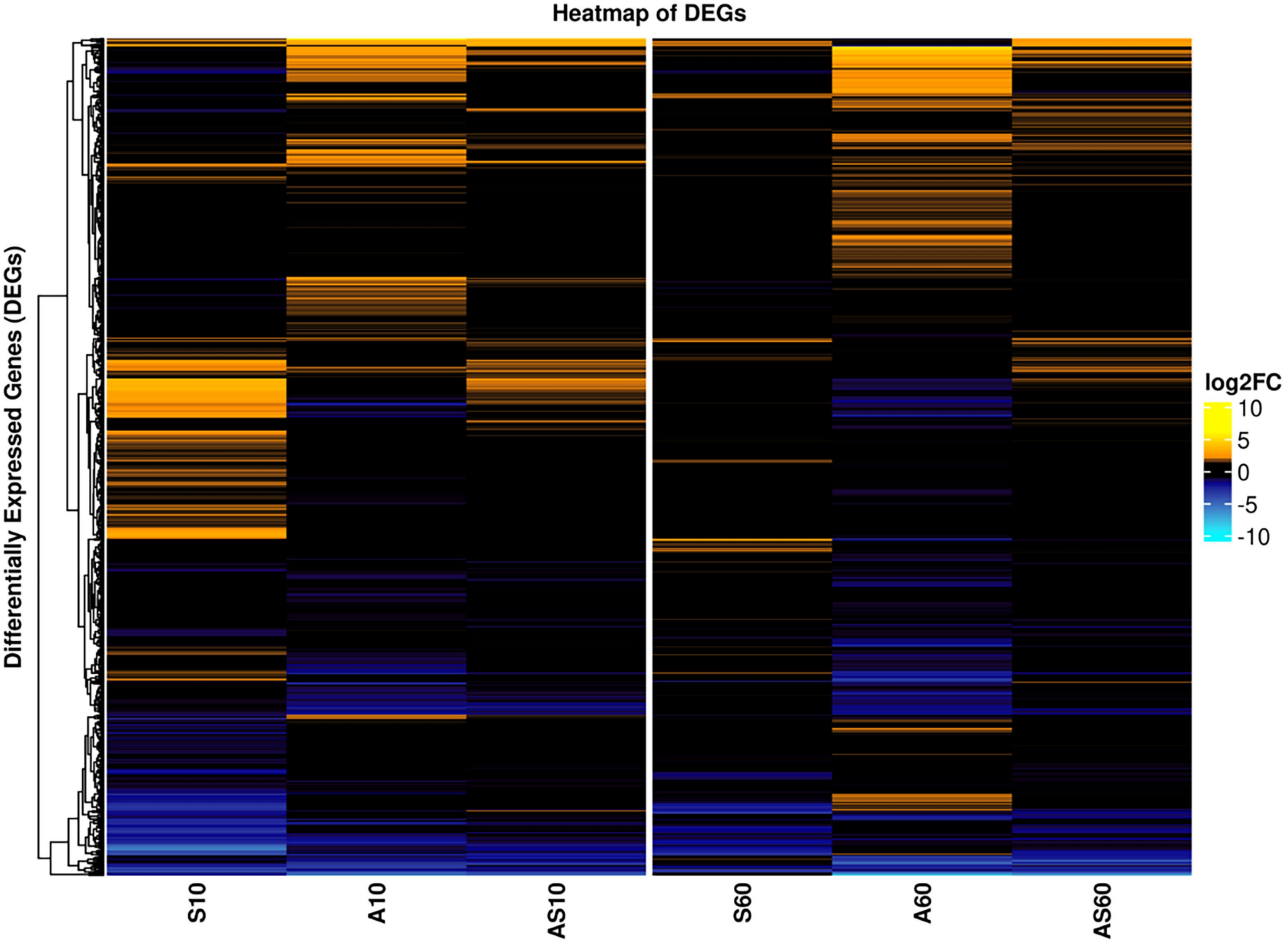
Global overview on differentially expressed genes for samples and conditions after 10 and 60 min of exposure. Pattern analysis of differentially expressed genes (DEGs) are visualized based on their log2 fold-change (log2FC) compared to the corresponding control samples, ranging from up- (yellow) to downregulated (blue). Each column corresponds to a distinct growth condition (S = serum, A = ammonia, AS = ammonia & serum) and time point (10 min and 60 min of exposure). Genes are clustered based on their respective expression across the tested conditions (left). Expression ranges from-10 log2FC to +10 log2FC.

**Figure 5:**
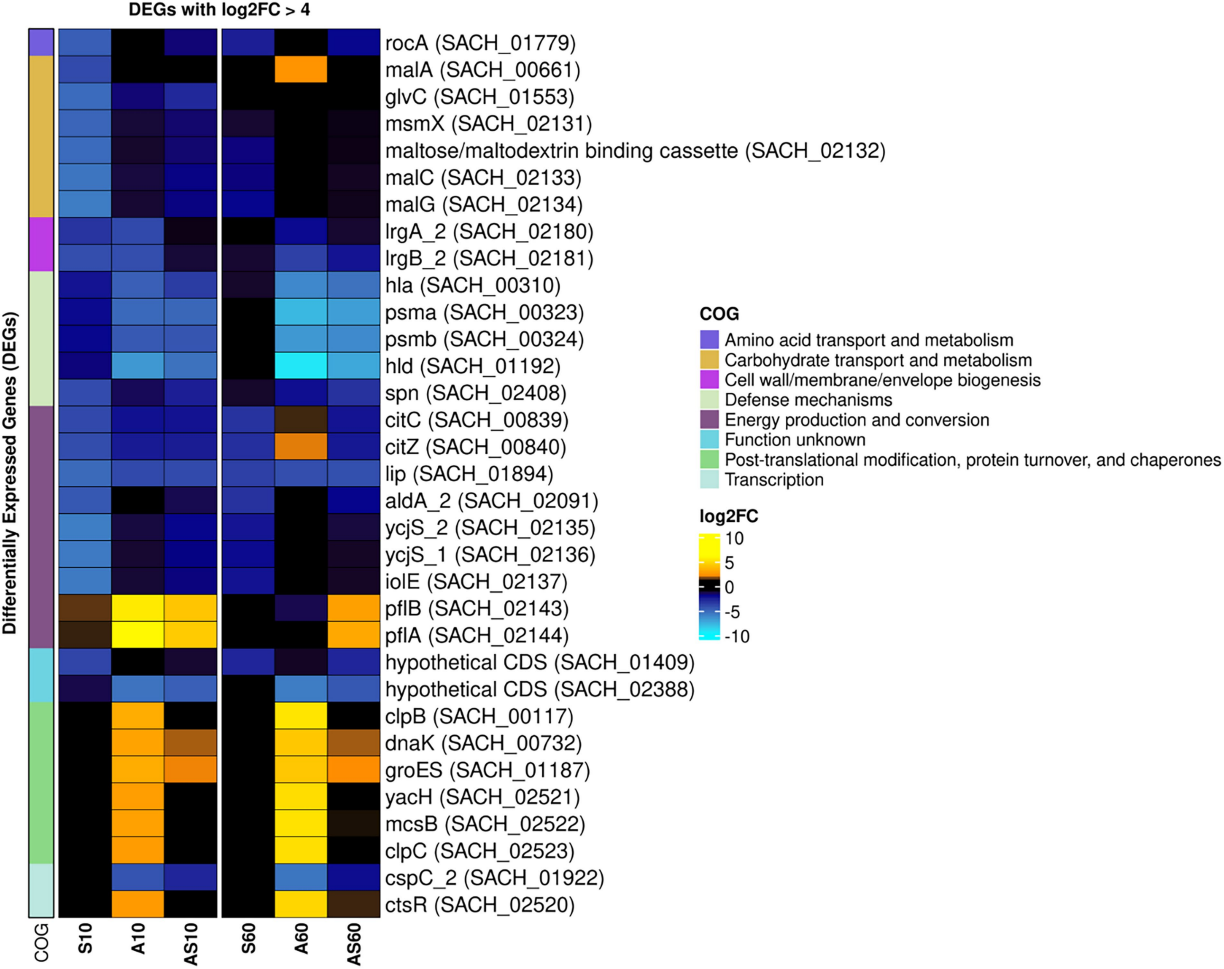
Heatmap of the top differentially expressed genes across all sample conditions. Top differentially expressed genes (DEGs) (n = 41) are visualized based on their respective log2 fold-change (log2FC) compared to the corresponding control samples. Each column corresponds to a stress condition (S = serum, A = ammonia, AS = ammonia & serum) and time point (10 min and 60 min). Genes are either strongly upregulated with log2FC > 5 (yellow) or downregulated with log2FC < −4 (blue). Genes are furthermore annotated with corresponding COG ontologies (left side).

The transcriptomic profiles of IMT38951 triplicate samples challenged by environment-mimicking conditions such as an increased ammonia concentration (pH 9.0), porcine serum (pH 7.3) and a combination of them (pH 8.9) were compared to corresponding unchallenged control samples (pH 7.2) after 10 and 60 min of exposure. Cell viability and growth kinetics were assessed from immediate exposure to 90 min afterwards (Supplementary Figure 2). In addition, exposure to each of the environment mimicking conditions seemed to require a characteristic cellular response, as demonstrated by the principal component analysis carried out to ensure limited variation between individual samples (Supplementary Figure 3). A global and summarized overview on gene expression in transcripts per million (TPM) and DEGs (corrected *p* ≤ 0.05), is provided in Supplementary Table 2. DEGs were divided into upregulated (log2FC > 1) and downregulated genes (log2FC < −1), revealing characteristic transcription patterns for each sampling time point and growth condition (global overview provided in Figure 4). Based on the heatmap revealing the highest differentially expressed genes across all sample conditions investigated (Figure 5), the most important mechanisms and pathways involved in transcriptomic responses have been further evaluated.

#### Global regulators

Global regulators that might be affected by the growth conditions were the class I heat shock responses comprising the HrcA regulon (*hrcA-grpE-dnaK-dnaJ* and *groESL*) that is embedded within the CtsR regulon (Michel et al., 2006) which was mostly induced, especially in the presence of ammonia (Table 1).

**Table 1:** Transcription of genes associated with coping mechanisms in livestock-associated MRSAST398.

				Gene transcription (log2FC) compared to unchallenged control samples					
				**Porcine serum**		**Ammonia**		**Porcine serum & ammonia**	
ID	**Symbol**	**Product**	**AA length**	**10 min**	**60 min**	**10 min**	**60 min**	**10 min**	**60 min**
SACH_00117	*clp*B	ATPase subunit of an ATP-dependent protease ClpB	870	0.0	−0.1	3.1	5.1	0.9	0.6
SACH_00140	*yjb*H	Disulfid stress effector	269	−0.4	−0.2	0.8	1.0	0.3	0.3
SACH_00499	*cat*A	catalase	506	−1.9	−2.1	2.9	2.7	1.2	0.9
SACH_00731	*dna*J	chaperone protein DnaJ	380	0.9	0.1	2.5	3.2	1.3	1.1
SACH_00732	*dna*K	molecular chaperone DnaK	611	0.3	0.1	2.8	4.1	1.7	1.7
SACH_00733	*grp*E	heat shock molecular chaperone protein	209	0.8	0.7	2.5	3.8	1.6	1.9
SACH_00734	*hrc*A	heat-inducible transcription repressor HrcA	326	0.8	0.3	2.4	3.7	1.6	1.7
SACH_01186	*gro*EL	co-chaperonin GroEL	539	−0.5	0.5	2.8	3.9	1.7	1.8
SACH_01187	*gro*ES	co-chaperonin GroES	95	−0.2	0.9	3.1	4.1	1.9	2.0
SACH_01418	*asp*23	Alkaline shock protein 23	170	−1.9	−1.3	2.4	0.2	1.3	−0.3
SACH_01786	*crt*N	dehydrosqualene desaturase	503	−0.8	−0.9	1.9	1.3	0.6	−0.1
SACH_01787	*crt*B	squalene/phytoene synthase	288	−0.8	−0.7	1.8	0.8	0.8	0.1
SACH_01788	*crt*Q	glycosyl transferase family protein	376	−1.1	−1.1	1.7	0.1	0.5	−0.1
SACH_02361	*ahp*F	alkyl hydroperoxide reductase large subunit	508	−1.9	−1.1	1.5	2.0	0.7	1.3
SACH_02362	*ahp*C	alkyl hydroperoxide reductase subunit C	190	−1.3	−0.9	1.9	2.7	1.0	1.8
SACH_02520	*cts*R	class III stress genes transcriptional repressor	157	−0.7	0.6	2.5	4.6	1.0	1.2
SACH_02521	*mcs*A	putative modulator of heat shock repressor CtsR (UvrB/UvrC motif-containing protein)	189	−0.6	0.2	2.6	4.9	0.4	0.9
SACH_02522	*mcs*B	D-isomer specific 2-hydroxyacid dehydrogenase family protein	337	−0.2	0.5	2.7	5.0	1.0	1.1
SACH_02523	*clp*C	Clp protease ATP binding subunit	819	−0.6	0.4	2.5	4.9	0.7	0.9

Transcriptional changes after 10 min and 60 min of MRSA IMT38951 exposed to Mueller-Hinton (MH) II bouillon supplemented with 10% porcine serum, 0.3 ammonia or both, respectively. ID, sequence identifier; AA length, amino acid length.

The center of the regulatory network in *S. aureus* consists of quorum sensing through the accessory gene regulator (*agr*) system, which concerts virulence factor transcription and synthesis, beyond others (Novick et al., 1993; Gaupp et al., 2016). We noticed a decrease in transcription of the *agr* locus compared to the respective unchallenged control samples for all experiments supplemented with ammonia (up to −4.7 log2FC; Table 2), a result likely related to the reduced growth kinetics associated with these conditions (Supplementary Figure 2). The addition of ammonia reduced the activity of the response regulator of the *agr* locus, AgrA. There was also down-regulation of AgrA-dependent genes/operons such as the phenol soluble modulins (PSMs) encoding operon (*psma* and *psmb*), as well as of the *agr*-inherent effector molecule RNAIII (a larger sRNA which also includes the delta-hemolysin open reading frame (*hld*)) noticed in ammonia-challenged cells (Supplementary Table 2). Reduced levels of RNAIII, are likely to decrease the transcription of toxin encoding genes. This is exemplified by decreased transcript levels of the α-toxin encoding gene *hla* (Supplementary Table 2), presumably *via* reduced degradation of *rot* transcripts, encoding the repressor of toxins (Boisset et al., 2007), which were found to be enhanced by 1.4 to 1.9 log2FC in presence of ammonia, as compared to the controls (Table 2). Reduced RNAIII levels may also explain the decreased transcript levels seen for *mgrA* (encoding the global transcriptional regulator MgrA) in presence of ammonia (Table 2), as RNAIII is known to stabilize the *mgrA* mRNA (Gupta et al., 2015).

**Table 2:** Transcriptional changes of global regulators in livestock-associated MRSAST398 exposed to ammonia porcine serum or both.

				Gene transcription (log2FC) compared to unchallenged control samples					
				Porcine serum		Ammonia		Porcine serum & ammonia	
ID	Symbol	Product	AA length	10 min	60 min	10 min	60 min	10 min	60 min
SACH_00575	*arl*S	signal transduction histidine kinase	452	−0.4	−0.1	−0.6	−1.8	−0.4	−0.9
SACH_00576	*arl*R	response regulator receiver	220	−0.6	−0.2	−0.7	−1.6	−0.6	−0.9
SACH_00645	*srrB*	integral membrane sensor signal transduction histidine kinase	584	0.1	0.2	−0.4	−0.6	0.6	0.2
SACH_00646	*srr*A	winged helix family two component transcriptional regulator	242	−0.2	0.4	−0.6	−1.1	0.8	0.5
SACH_00713	*rpo*D	RNA polymerase sigma-70 factor RpoD (SigA)	369	0.9	0.1	0.3	−0.1	0.7	0.7
SACH_00905	*rot*	repressor of toxins (Rot)	134	−0.3	−0.1	1.6	1.9	1.5	1.4
SACH_00917	*sig*S	RNA polymerase factor sigma-70	157	1.0	0.5	−0.3	−0.4	−0.2	0.1
SACH_01193	*agr*B	accessory gene regulator B	208	−0.9	−0.4	−2.1	−3.7	−1.8	−4.7
SACH_01194	*agr*D	staphylococcal accessory gene regulator protein D	47	0.3	0.5	−1.6	−3.4	−1.2	−4.5
SACH_01195	*agr*C	accessory gene regulator protein C	431	−0.6	−0.5	−2.1	−4.0	−1.9	−4.6
SACH_01196	*agr*A	autoinducer sensor protein response regulator protein	239	−0.9	−0.5	−2.5	−3.7	−2.1	−4.2
SACH_01284	*sig*B	RNA polymerase sigma factor SigB	257	0.4	−0.3	0.4	−1.3	1.3	0.3
SACH_01524	*sar*R	staphylococcal accessory regulator R SarR	116	0.0	0.5	−1.9	−4.1	−2.8	−2.2
SACH_02036	*sar*S	staphylococcal accessory regulator A SarS	251	0.3	0.4	−1.0	−0.9	0.0	0.6
SACH_02532	*sig*H	RNA polymerase factor sigma-70	190	0.7	0.2	1.2	0.4	0.9	0.9
SACH_02614	*sar*A	staphylococcal accessory regulator family protein	125	0.9	−0.5	1.5	−0.4	1.1	−0.1
SACH_02686	*mgr*A	MarR family transcriptional regulator	148	−0.4	0.1	−1.4	−2.7	−1.4	−0.5
SACH_02705	*sae*S	integral membrane sensor signal transduction histidine kinase	352	−0.3	0.7	−1.6	−3.3	−2.3	−2.6
SACH_02706	*sae*R	winged helix family two component transcriptional regulator	229	−0.1	1.0	−1.5	−3.1	−2.1	−2.4

Transcriptional changes after 10 min and 60 min of MRSA IMT38951 exposed to MH II supplemented with 10% porcine serum. 0.3 ammonia or both, respectively. ID, Sequence identifier; AA length, amino acid length.

When IMT38951 cultures were challenged with ammonia (in presence or absence of serum), clear reductions in *sae* (*S. aureus* exoprotein expression) transcript rates were noticed for both time points monitored (Table 2). Another potential factor contributing to the decrease in *sae* transcripts seen in presence of ammonia is SigB. This stress-induced alternative sigma factor is known to decrease *sae* transcription (Geiger et al., 2008), and is activated among others by a rapid increase in pH (Pané-Farré et al., 2009). Although the *sigB* operon is partially autoregulated, SigB activity is best followed by monitoring alkaline shock protein 23 (*asp*23) transcription (Giachino et al., 2001). Here, the sigma-B regulated *asp*23 gene (Kuroda et al., 1995) was induced after 10 min of exposure to ammonia alone (2.4 log2FC) or the combination of ammonia and porcine serum (1.2 log2FC; Table 1). In addition, transcription of only a part of the SarA regulon was found to be altered by ammonia supplementation in a relevant manner (i.e., *hla*, *hlg*A, *lrg*AB, *nrd*G, *psma*, but not *aur*, *esx*A, *ica*ADBC, *nuc*, *sdr*D, and *sod*M; Supplementary Table 2).

#### Presence of reactive oxygen species/protein synthesis

Although the equilibrium of the chemical reaction NH_3_ + H_2_O ⇌ NH4^+^ + OH-shifts towards the left site (especially at pH 9), the rather limited number of free protons in bacterial cells (Saito and Kobayashi, 2003) may be captured by the hydroxyl radicals (OH^−^). Therefore, the formation of a small fraction of hydroxyl radicals (OH^−^) in all samples exposed to ammonia is expected (Saito and Kobayashi, 2003). Here, we noted a transient induction of genes (maximum at 10 min of exposure) known for their response to the presence of reactive oxygen species (ROS) such as hydroxyl radicals (Pandey et al., 2019) including parts of the machinery involved in staphyloxanthin biosynthesis such as the *crtOPQMN* operon (Pelz et al., 2005), a carotenoid thought to protect *S. aureus* from oxidative stress (Clauditz et al., 2006). Similarly, genes encoding catalase (*kat*A) and especially alkyl hydroperoxide reductase (*ahp*C) were most effectively induced after 10 & 60 min of exposure to ammonia alone (Table 1).

Exposure to serum or even the combination of serum and ammonia did not affect transcription of the gene encoding the ribosome-associated translation inhibitor (*rai*A) after either 10 or 60 min, although 60 min of exposure to ammonia alone lead to a 2.0 log2FC, indicating the necessity to decrease overall protein production to cope with the presence of enhanced ammonia concentrations for more than an hour (Supplementary Table 2).

#### Virulence/defence

Of note, transcription of several virulence-and defence associated genes was transiently induced by exposure of MRSA ST398 to porcine serum only, i.e., genes encoding the von Willebrand-factor binding protein (10/60 min 2.2/1.4 log2FC), the chromosomal staphylococcal complements inhibitor (*scn*; 10/60 min 1.0/0.2 log2FC), an additional pathogenicity island-associated *scn* variant (10/60 min 1.5/1.6 log2FC), staphylocoagulase (*coa*; 10/60 min 2.2/0.6 log2FC) and immunoglobulin G-binding protein (*sbi*; 10/60 min 1.1/0.7 log2FC; Supplementary Table 2).

### Transcriptional response of genes associated with SOS responses and horizontal transfer of SCC*mec*

**Figure 6:**
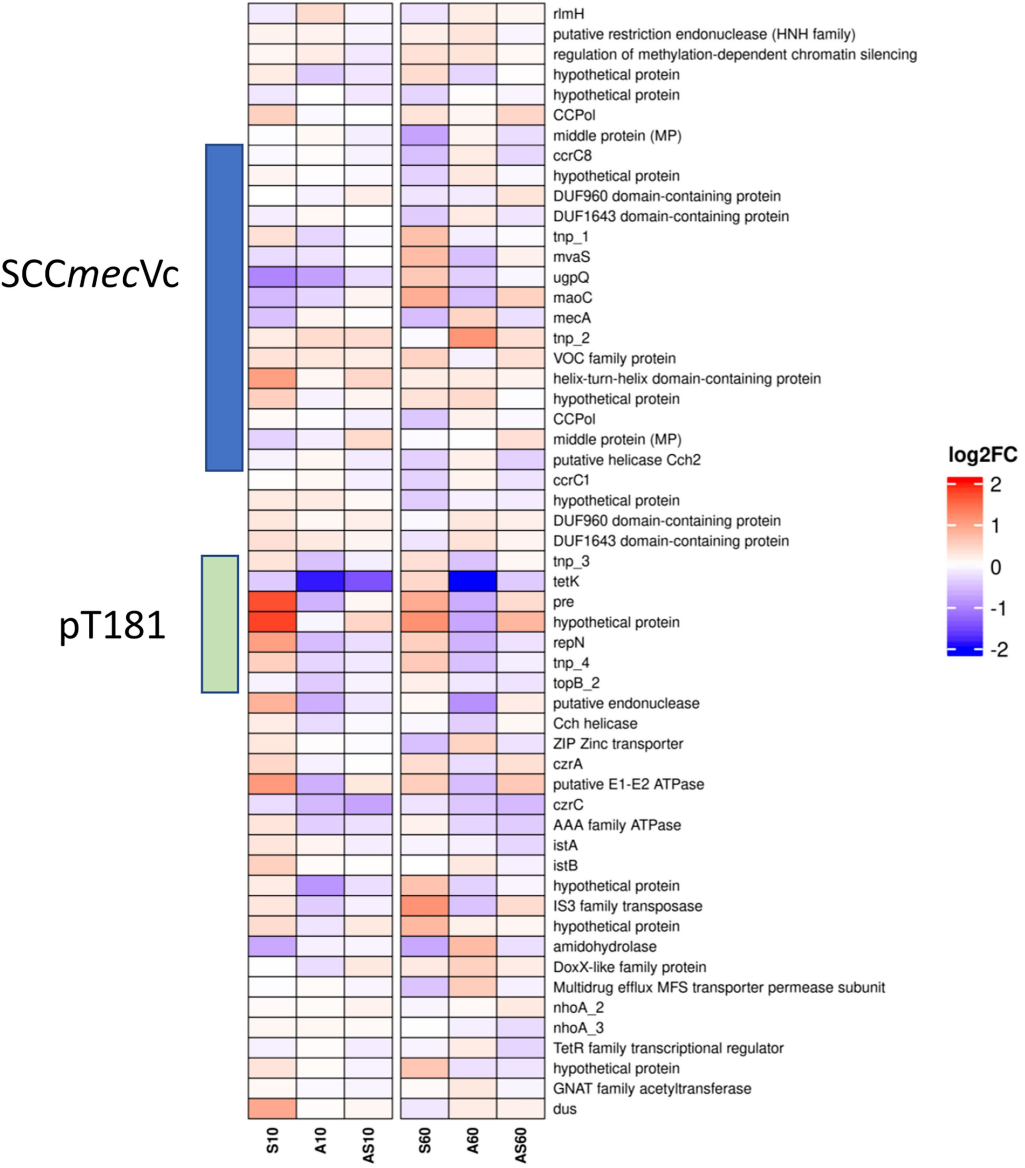
Heatmap illustrating transcription of genes at the chromosomal SCC*mec* integration site from *rlm*H (“*orf*X”) to *dus* (“*orf*Y”). Gene transcripts are visualized based on their log2 fold-change (log2FC) with either red (upregulated), blue (downregulated) or white (no changes) compared to the corresponding control samples. Each column corresponds to a growth condition (S = serum, A = ammonia, AS = ammonia & serum) and time point (10 min and 60 min). Overall expression ranges from-2 log2FC to +2 log2FC.

In *S. aureus*, the SOS response following the occurrence of ssDNA is tightly regulated by the interplay of the DNA damage-inducible repressor LexA and the recombination protein RecA (Butala et al., 2009; Maslowska et al., 2019; Podlesek and Žgur Bertok, 2020). Activation of RecA by binding to single-stranded DNA promotes transcriptional upregulation of genes belonging to the SOS-regulon by activation of LexA self-cleavage from the respective promoter sites. Although actual activation of the synthesized proteins cannot be followed by transcriptional analysis, some of the genes associated with SOS response in *S. aureus* are transcriptionally induced, i.e., the topoisomerase IV genes *par*E and *par*C at 10 and 60 min (1.3 /0.6 and 1.7 /0.8 log2FC) after exposure to porcine serum (Supplementary Table 2). The LexA-regulated low-fidelity and error-prone DNA polymerase V (Ha and Edwards, 2021) *umu*C (1.7 log2FC) and genes involved in nucleotide excision repair (*uvr*A/B; 1.5/1.2 log2FC) are significantly induced after 60 min of exposure to ammonia. Since a similar SOS-mediated induction of *ccr* genes by cleavage of LexA from its promoter sequence was reported previously (Liu et al., 2017), the transcriptional response of both *ccr* variants was evaluated. Both *ccr* allelic variants in IMT38951 lacked significant induction over the time points and conditions evaluated here, although a minimal induction was noted after 60 min of exposure to ammonia (between 0.1 and 0.2 log2FC; Figure 6). However, this observation seems in overall congruence with the limited but notable induction of genes belonging to the SOS-response after 60 min of exposure to ammonia described above. The transcription of *lex*A, on the other hand, is 0.7 log2FC after 10 and 1.5 log2FC after 60 min of exposure to ammonia, a rate that may indicate that LexA has been consumed by SOS-induction. Of note, *rec*A transcription levels lack significant changes compared to the unchallenged controls at all conditions investigated (Supplementary Table 2).

Data on up-or downregulation of predicted CDSs within the genomic region harboring SCC*mec*, starting with the rRNA large subunit methyltransferase (*rlm*H) and ending with tRNA dihydrouridine synthase (*dus*), is provided in Figure 6 and Supplementary Table 2 (highlighted in green). Considering genes putatively involved in mobilization and recombination of transferrable MGEs present in this particular region, we noticed an increased transcription of transposase *tnp*_2 associated with IS431, located adjacent to *mec*A, after 10 min (0.3 log2FC) and 60 min (1.0 log2FC) of exposure to ammonia (Figure 6). Moreover, the replication initiation gene *rep*N (10/60 min 1.0/0.5 log2FC) and the gene encoding for a plasmid recombination protein (*pre*; 10/60 min 1.7/0.8 log2FC), both belonging to the tetracycline resistance gene (*tetK*)-carrying plasmid pT181 that integrated into the SCC*mec* region downstream of *ccr*C1 (Figure 3A), showed a transcriptional increase after exposure to porcine serum (Figure 6; Supplementary Table 2).

Genes associated with both variants (I and II Figure 3) of the replication machinery CCPol-MP-Cch2 were not significantly induced by the growth conditions investigated here (Supplementary Table 2; Figure 6).

## Discussion

### Reconstruction of the loss of methicillin resistance in IMT38951_42 revealed deletion of a complete and putatively replicative SCC*mec*Vc element by homologous recombination

Following the loss of methicillin resistance in IMT38951 genomic investigations were hindered by multiple repetitive sequences in the region downstream of *rlm*H. This was overcome by a combination of long read sequences polished by short reads to allow for an accurate genomic inspection of this particular region, including a detailed reconstruction of the process most likely leading to the loss of the complete SCC*mec* element from its chromosomal integration site, outlined in Figure 3.

Previous research indicated that deletion followed by HR, or excision driven by large serine recombinases, allow complete SCC*mec* elements to detach from their chromosomal integration site downstream of *rlm*H (Hanssen and Ericson Sollid, 2006; Chlebowicz et al., 2010; Wang and Archer, 2010; Liu et al., 2017; Gómez et al., 2020). This likely requires the involvement of the serine recombinases (*ccr*A/B or *ccr*C) that facilitate chromosomal integration and excision of SCC*mec,* or other factors belonging to the bacterial recombination machinery. Since SCC*mec* elements are typically flanked by “direct repeats” (DR) of 15 bp length that are recognized by the respective large serine recombinases, *ccr*-driven integration or loss of these elements always leads to changes in the number of DRs at the chromosomal integration site (Ito et al., 1999; Semmler et al., 2016). However, since the number and position of the two direct repeats present in the MRSA predecessor remained unchanged in the descendent, MSSA IMT38951_42, *ccr*-mediated excision of the SCC*mec* element, as described previously (Noto et al., 2008; Wang and Archer, 2010; Zhang et al., 2015, 2016), is unlikely.

A further study identified recombination between the putative primase-encoding (now: helicase Cch2, according to Bebel et al., 2020) genes belonging to the CCPol-MP-Cch2 complex in the ST398 lineage (Gómez et al., 2020). Other authors have linked the loss of SCC*mec* with recombination between *ccr*C allelic variants (Chlebowicz et al., 2010; Vandendriessche et al., 2014), a possibility that cannot be ruled out for the isogenic pair belonging to ST398 in our study (Figure 3, recombination between B and C). However, the actual region prone to recombination described above comprises the end of intergenic spacer region 1 and the initial 48 bp of both *ccr* genes (Figure 3A), a region that harbors four identical short repetitive sequences. Interestingly, both recombination events (A & C and B & D) events would produce an identical SCC*mec* circular element (Figure 3D). Consequently, occasional deletion followed by HR might be of greater biological importance for the spread of methicillin resistance than previously considered, as Bebel et al. reported autonomous replication capabilities for exactly these elements (Bebel et al., 2020) that contribute not only to the maintenance of the circular SCC*mec* intermediates, but also to their proliferation within an extra-chromosomal space. Subsequently, novel SCC*mec* elements might be available for horizontal transfer to other suitable recipients - a hypothesis that should be addressed in future research.

### *In vitro* transcriptional response to environmental challenges associated with pig farming highlights the adaptive capabilities of MRSA ST398

Recently, the ability of the MRSA ST398 lineage to survive in pig manure has been demonstrated (Astrup et al., 2021). This particular LA environment is known for high natural ammonia levels - a major selective factor for bacterial community structures (Nordgård et al., 2017). In *S. aureu*s, early responses to environmental challenges are often associated with changes in the activity of global regulators (Crosby et al., 2016; Ranganathan et al., 2020; Párraga Solórzano et al., 2021). Activation of components belonging to the heat shock response seems to be among the most important coping mechanisms involved with respect to ammonia-rich environments. The latter is a protective mechanism considered crucial for bacterial survival and adaptation to hostile environmental conditions by degradation of misfolded and denatured proteins and prevention of protein misfolding events (Roncarati and Scarlato, 2017).

The number of free protons present in a bacterial cell depends on the pH: At an alkaline pH above 9, the number of free protons is reduced to less than one per bacterium (calculated for *Escherichia coli*), a fact that hinders the protonation of specific sites in enzymes required for their activity (Saito and Kobayashi, 2003). Consequently, cellular stress including disturbances of pH-sensitive enzymatic reactions, energy metabolism and secondary protein structures were expected to feature in the transcriptomic responses of *S. aureus* cells exposed to ammonia at an alkaline pH (Gutierrez et al., 2022). Moreover, the data suggests that the electron pair of the NH_3_ molecule might interfere with hybrid bonds of peptides and/or proteins under these conditions, since addition of more protein (i.e., presence of porcine serum) reduces the effects associated with exposure to ammonia alone, arguing against a pH dependent effect alone. In line with this, the initial pHs of the ammonia-supplemented samples differed only slightly (ammonia: pH 9.0/ammonia & porcine serum: pH 8.9) and did not change significantly over time.

A reduction of the bacterial transcription machinery prevents excessive energy loss from protein synthesis (Roncarati and Scarlato, 2017). As shown in Supplementary Figure 2, viable cell numbers had the smallest increase over time in samples exposed to ammonia only, suggesting that down-regulation of the translation machinery (Supplementary Table 2) contributes to survival and adaptation in ammonia rich environments.

In addition, the *asp*23 expression that is controlled by the alternate sigma factor of RNA polymerase (SigB) was upregulated after 10 min of exposure to 0.3% ammonia and the combination of ammonia and porcine serum, an increase that has been reported for sodium hydroxide-induced pH increases previously (Anderson et al., 2010). However, after 60 min, the *asp23* transcription rates were comparable to the control, suggesting that ammonia supplementation induces a short-lived activation of SigB. This is in line with earlier observations reporting that SigB activity is only transiently induced by stresses such as heat shock (Gertz et al., 1999; Giachino et al., 2001). Further support for a stimulating effect of ammonia on SigB activity is given by our findings of increased transcript rates for several SigB-regulated genes known to be directly controlled by this factor such as the genes *crtMN,* whose products are involved in staphyloxanthin biosynthesis, a carotenoid thought to protect *S. aureus* from oxidative stress (Clauditz et al., 2006), *spoVG,* a transcription factor acting downstream of SigB (Meier et al., 2007), and *sar*A (Bischoff et al., 2001). Notably, SarA is also reported to act as regulatory protein responsive to redox and pH (Fujimoto et al., 2009), and to repress the expression of *rot* (Manna and Ray, 2007).

The observed reduction in *sae* transcription in all samples except those exposed to porcine serum for at least 60 min might be, at least in part, due to increases in SigB and Rot-activities, which were shown to decrease *sae* transcription from the *sae* P1 and P3 promoters, respectively (Geiger et al., 2008; Li and Cheung, 2008), and by a reduction in RNAIII transcripts, which are known to enhance *sae* transcription (Novick and Jiang, 2003; Geiger et al., 2008).

Although not directly regulated by *agr*, gene transcription of a few virulence factors located on mobile genetic elements that can be considered as a specific response to host (porcine) proteins such as *scn* and the von Willebrand Factor binding protein were decreased in all samples exposed to ammonia with or without porcine serum, indicating that the pH of 8.9–9.0 and/or the NH_3_ molecule interferes with (host-)protein recognition, requires energy saving or both.

Transcription of genes involved in capsular polysaccharide biosynthesis in ammonia supplemented media was not recorded, although their upregulation due to increased alkaline conditions has been demonstrated before (Anderson et al., 2010).

Taken together, LA-MRSA ST398 seems fully equipped to endure pig-farm associated environmental factors such as ammonia and porcine serum, helping to support their long-term survival within that environment.

### Transcription of genes involved in SC*Cmec* mobility

In *S. aureus*, the genomic region between origin-of-replication (*ori*) and the SCC generally seems prone to HR, with the integration site for SCC representing a particular “hotspot” in this regard (Everitt et al., 2014; Semmler et al., 2016). A recent study investigated the stability of four different SCC*mec* elements (I, II, III, IV) during 3 months of serial subculturing at room temperature, revealing that SCC*mec* stability was influenced both by internal mobile elements (IS431) as well as the cell environment (Scharn et al., 2022). Exposure to pig-farming associated environmental challenges did not significantly alter the expression ratios of genes likely to be involved in the mobilization of complete or partial SCC*mec* elements, including transposases associated with IS431 and the *ccr* recombinases (Wang and Archer, 2010; Wang et al., 2017; Scharn et al., 2022) in this study when compared to unchallenged controls. These results suggest that the aforementioned conditions do not strongly contribute to SCC*mec* mobility to a large extent - at least not within the timeframe investigated here.

Moreover, exposure to ammonia, porcine serum or both failed to significantly induce *rec*A transcription that would lead to de-repression of LexA-controlled genes including allelic variants of *ccr*C, as reported before (Liu et al., 2017). Although some genes tightly regulated by LexA such as *uvr*AB and *umu*C showed a clear log2FC increase after 60 min of exposure, the corresponding transcription rate noted for both *ccr* genes (0.1/0.2 log2FC) lacked significant difference compared to the controls. Since autocatalysis of LexA is required for derepression of some genes within the SOS regulon (Ha and Edwards, 2021) including *lex*A, others are only derepressed when the N-terminal domain is further digested by Clp proteases including ClpCP. Here we noted an induction between 2.5 log2FC after 10 min and 4.9 log2FC after 60 min for *clp*C in cells exposed to ammonia only (Table 1), once more suggesting a fine-tuned activation of the SOS machinery in MRSA ST398 strain IMT38951. Therefore, a massive cleavage of LexA from the *ccr*C promoter region may depend on more invasive DNA damaging conditions, i.e., the presence of distinct antibiotics, as reported before (Cirz et al., 2007; Liu et al., 2017). It seems worth considering that DNA-damaging effects attributed to ammonia rich environments and further challenging circumstances, for instance, exposure to antibiotics, may accumulate *in vivo*. In light of the recent results of Scharn et al. (2022), we speculate that prolonged exposure to livestock-associated conditions might induce a more prominent transcription of the *ccr* genes.

## Conclusion

Here we followed the deletion of a complete and fully functional SCC*mec* Vc element from its chromosomal integration site downstream of *rlm*H in an MRSA ST398 isolate (IMT38951) and highlighted the most important transcriptional responses of the bacteria required to endure pig-farm associated environmental challenges such as increased ammonia concentrations and porcine serum. A detailed reconstruction of SCC*mec* loss from its genomic integration site raises concerns with respect to the autonomous replication capabilities of the predicted circular SCC*mec* element and its potential to spread to other susceptible bacterial hosts. It seems likely that this particular method of SCC*mec* conservation and transmission might have been underestimated, especially considering that, at least so far, only severe DNA-damaging events seem to trigger an increased activity of the *ccr*C variants at the population level. However, further studies are required to evaluate which factors and co-factors may trigger and support the autonomous replication machinery present in the excised elements.

## Data availability statement

The datasets presented in this study can be found in online repositories. Raw sequencing reads, including RNA-Seq and long-read genomic data, were uploaded to NCBI and deposited within BioProjects PRJNA891722 and PRJNA449454.

## Author contributions

CH, AL-B, LW, LE, SW, and BW contributed to conception and design of the study. CH, JB, JA, and BW performed laboratory experiments and analysis. Genomics and transcriptomics were carried out by LE, SW, AT, and TS. CH, MB, MH, AB, WZ, and BW analysed the results. CH and BW wrote the first draft of the manuscript. LE, SW, WZ, and MB wrote sections of the manuscript. All authors contributed to the article and approved the submitted version.

## Funding

This work was supported by the German Federal Ministry of Education and Research (BMBF) for #1Health-PREVENT (grant nos. 01KI2009D and 01KI2009F) and PAC-CAMPY (grant no. 01KI2007F) within the German Research Network of Zoonotic Diseases. The funders had no role in study design, data collection and analysis, decision to publish, or preparation of the manuscript. CH received a research doctoral fellowship from Akademie für Tiergesundheit e.V. (AfT; Germany).

## Conflict of interest

The authors declare that the research was conducted in the absence of any commercial or financial relationships that could be construed as a potential conflict of interest.

## Publisher’s note

All claims expressed in this article are solely those of the authors and do not necessarily represent those of their affiliated organizations, or those of the publisher, the editors and the reviewers. Any product that may be evaluated in this article, or claim that may be made by its manufacturer, is not guaranteed or endorsed by the publisher.

## Footnotes

^1^ https://www.biosearchtech.com/services/
