# Supplementary material for: How to survive pig farming: Mechanism of SCCmec element deletion and metabolic stress adaptation in livestock-associated MRSA
Source: Front Microbiol. 2022 Nov 23;13:969961. doi: 10.3389/fmicb.2022.969961 (PMC9728531; doi:10.3389/fmicb.2022.969961)
Supplement: Supplementary file 1 [file Data_Sheet_1.PDF]

Supplementary Figure 1| Comparison of intergenic spacer regions (ISR1 and ISR2), *ccr*C8 and *ccr*C1 (first 48 bp each)

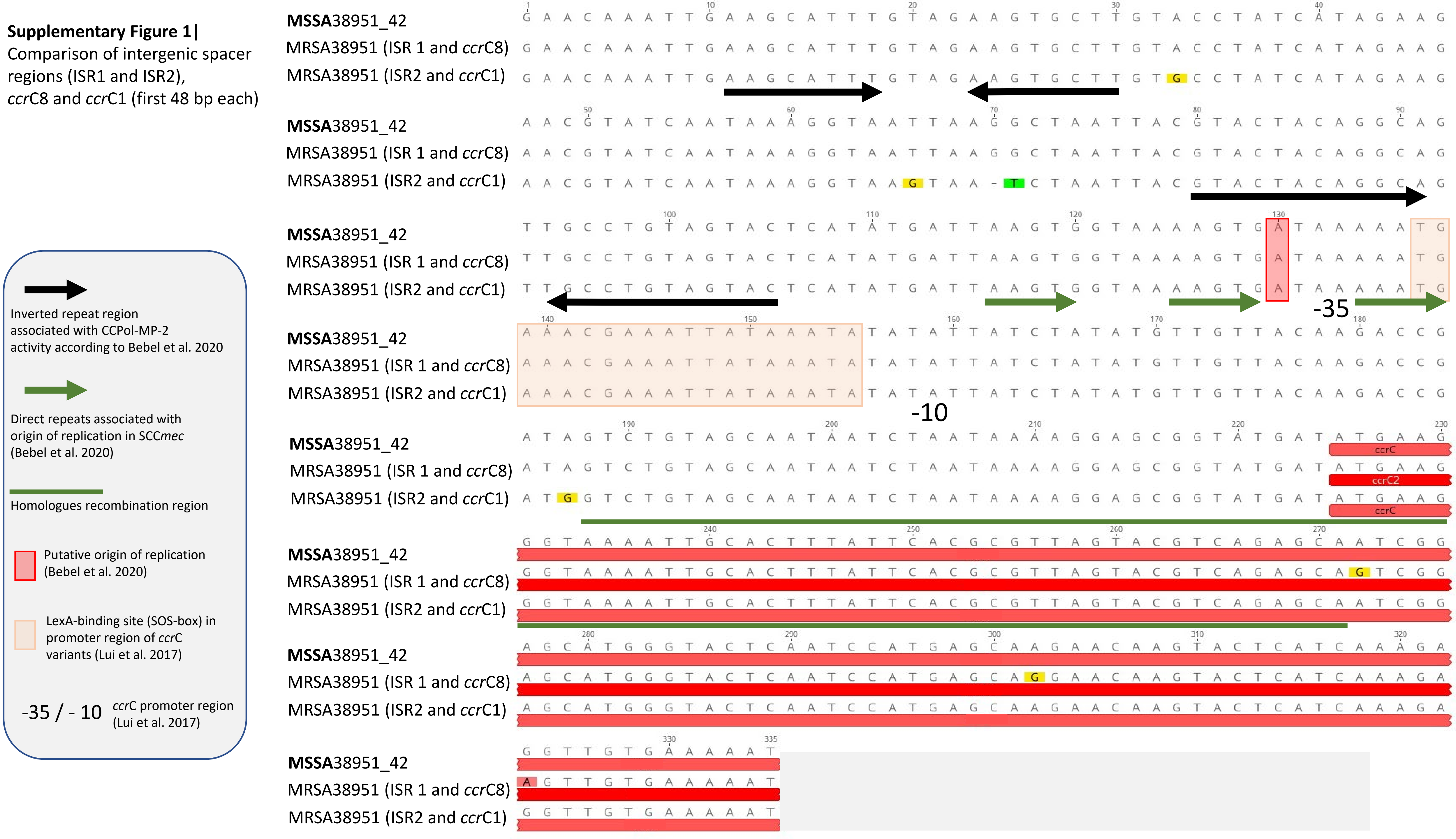

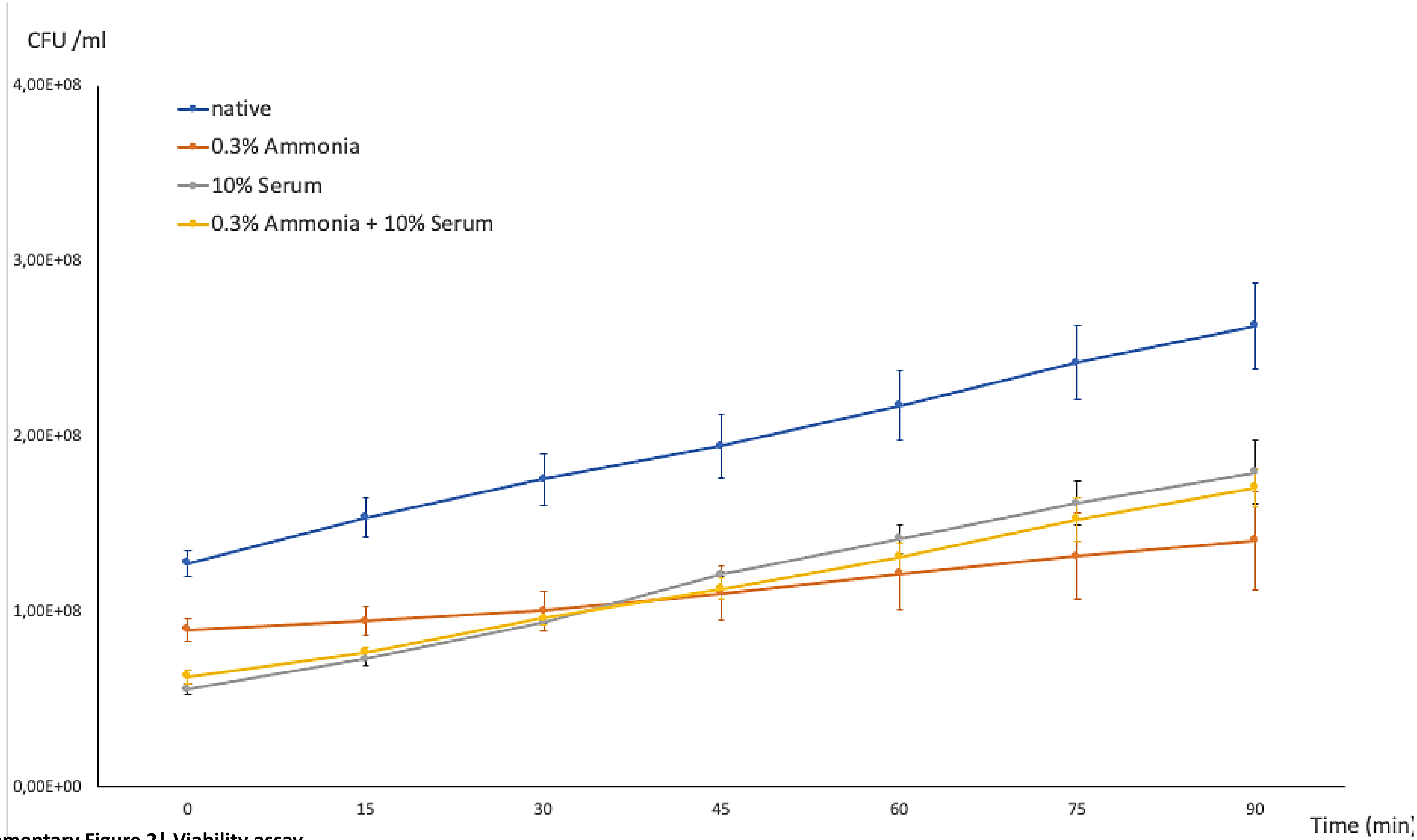

Supplementary Figure 2| Viability assay

Recovered MRSA IMT38951 are shown as colony forming units (CFU) per ml on a log scale (start: 0.4 OD_{600}). Bacteria were challenged by different supplements mimicking pig-farming environmental conditions such as enhanced ammonia, porcine serum and a combination of both for 90 min. Three independent experiments in technical triplicates are shown.

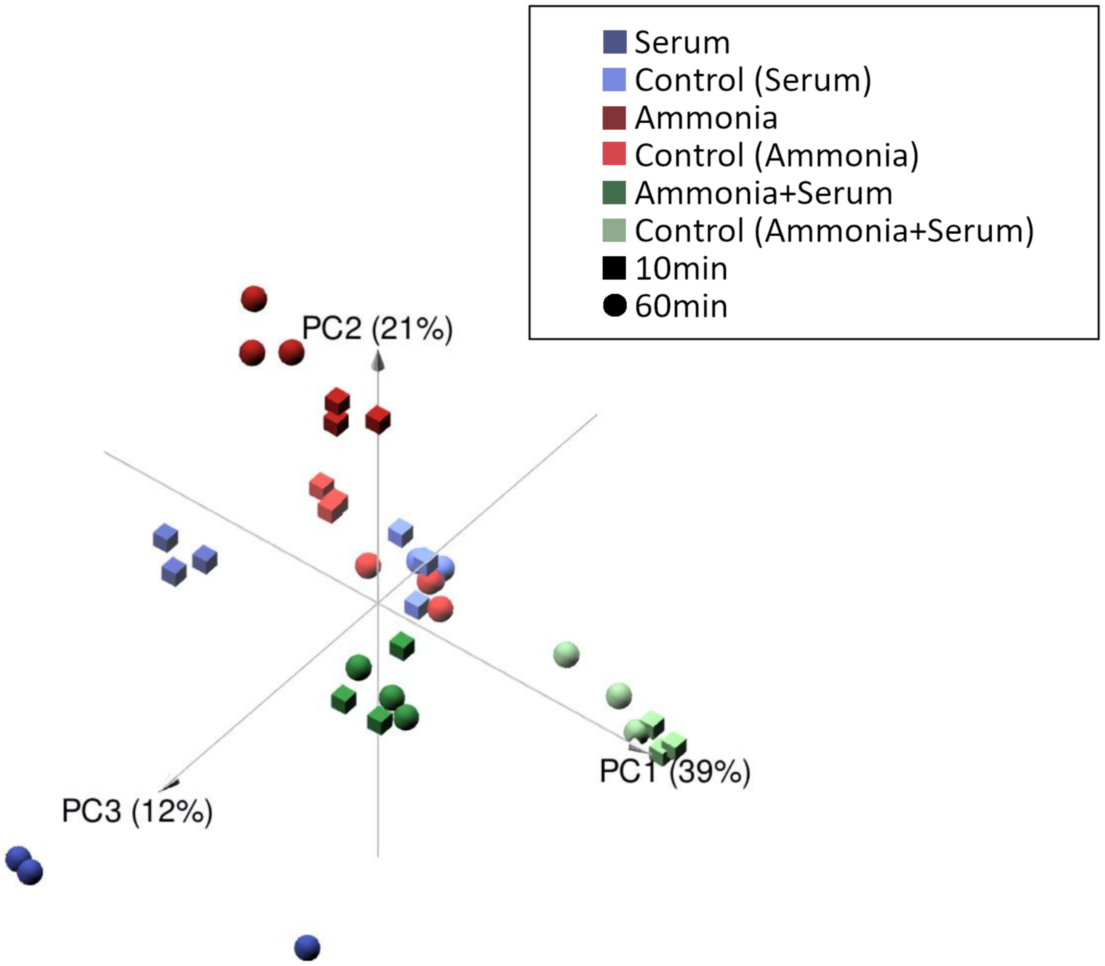

Supplementary figure 3| Principal Component Analysis (PCA) of transcription levels between samples. Inter-sample distances were calculated based on the transcripts per million (TPM) expression values of their respective gene content. PC1 (39%), PC2 (21%) and PC3 (12%) were used to visualize the similarity of the samples in a three-dimensional space. Samples were color-coded according to the distinct exposure conditions (serum = blue, ammonia = red, ammonia & serum = green) and different symbols illustrate the corresponding time points (ball = 10min, cube = 60min).
